# Microbial diversity, culture conditions, and application effect of YSJ: A composite microbial system for degradation of Yanshan ginger branches and leaves

**DOI:** 10.1371/journal.pone.0278701

**Published:** 2022-12-02

**Authors:** Wenhao Chen, Juan Li, Huawei Yuan, Ling You, Tao Wang, Zongjin Cao

**Affiliations:** 1 Faculty of Agriculture, Forestry and Food Engineering of Yibin University, Yibin, Sichuan Province, China; 2 Solid-State Fermentation Resource Utilization Key Laboratory of Sichuan Province, Yibin, China; 3 Sichuan Oil Cinnamon Engineering Technology Research Center, Yibin, China; 4 Faculty of Economics and Business Administration of Yibin University, Yibin, Sichuan Province, China; University of Jeddah, SAUDI ARABIA

## Abstract

**Background:**

Yanshan ginger (*Alpinia zerumbet*) is a perennial herb used as a medicine and spice, and is beneficial for soil and water conservation in karst areas. Given the widespread utilization of Yanshan ginger in China and continuing expansion of the planting area, disposal of waste materials is problematic. The branches and leaves of Yanshan ginger contain a variety of potent antibacterial compounds, such as volatile oils, phenols, and diterpenoids, which hinder their rapid degradation by microorganisms. In this study, we screened and constructed a composite microbial system to provide a technical reference for production of organic fertilizer from the branches and leaves of Yanshan ginger.

**Methods:**

A composite microbial system, “YanShan Jun” (YSJ), was developed by screening for efficient detoxification and degradation of the branches and leaves of Yanshan ginger. High-throughput sequencing technology was used to investigate the stability and diversity of YSJ subcultures. The culture conditions for YSJ were optimized by sequential single-factor experiments and response surface analysis. Yanshan ginger leaves and branches were inoculated with YSJ to study its effects on composting efficiency.

**Results:**

The microbial composition of YSJ was stable and rich in diversity through continuous subculture. Through response surface analysis, the optimized culture conditions for YSJ were determined as follows: peptone 8.0 g/L, sodium chloride 9.0 g/L, calcium carbonate 5.2 g/L, yeast powder 1.6 g/L, cultivation temperature 56.1°C, and culture duration 6 d. Under these conditions, the degradation rate of Yanshan ginger was 58.32%, which was 14.22% higher than that before optimization. The ability of YSJ to degrade the antibacterial compounds of ginger after optimization was significantly enhanced. Inoculation of Yanshan ginger compost with YSJ increased the fermentation temperature, prolonged the high-temperature period, and reduced the water content and pH of the compost in the early stage.

**Conclusions:**

Inoculation of plant compost with YSJ bacteria improves the nutritional environment of the compost, promotes the composting reaction, promotes the rapid formation of a strong indigenous microflora, forms a beneficial microecological environment, and increases the composting efficiency. This study provides a theoretical basis for practical application of YSJ for organic fertilizer production from Yanshan ginger.

## Introduction

Yanshan ginger (*Alpinia zerumbet*) is a perennial herb that is used as a traditional medicine and spice, and is beneficial for soil and water conservation in karst areas [1]. Given the widespread utilization of Yanshan ginger in China and continued expansion of the planting area, the issue of disposal of ginger waste materials has become problematic. After the harvest of ginger fruit, it is necessary to cut off the branches to increase the fruit yield in the following season. No previous research on appropriate treatment methods for Yanshan ginger branches and leaves has been conducted [2–5]. The most effective approach in China is to mix the branches and leaves with livestock and poultry manure to make bio-organic fertilizer [6]. However, the greatest problem is that compost fermentation does not proceed smoothly. This is because the branches and leaves of ginger contain volatile oils, phenols, pyrones, flavonoids, diterpenes, and other compounds with strong antibacterial effects [7, 8]. Therefore, there is a need to construct a bacterial agent that can quickly detoxify the branches and leaves of Yanshan ginger for efficient fermentation and production of bio-organic fertilizer [9, 10].

Composite microbial systems consist of bacteria that can be cultured separately and those that cannot be cultured [11]. The bacteria can maintain growth and metabolic activities under the inhibition of the biotoxic substances of Yanshan ginger, and effectively degrade some of the biotoxic substances and cellulose [12]. Therefore, it is feasible to simulate natural conditions and exploit the interaction of multiple microorganisms to detoxify the branches and leaves of ginger [13, 14].

In this study, we screened and constructed a composite microbial system, designated “YanShan Jun” (YSJ), with the capacity to degrade branches and leaves of Yanshan ginger from samples of pig manure combined with Yanshan ginger organic fertilizer. The compound microbial agent was used to detoxify and degrade ginger branches and leaves, and the culture conditions were optimized. Our aim was to provide a technical reference for production of an organic fertilizer from the branches and leaves of Yanshan ginger in combination with livestock manure in the future [15–17].

## Materials and methods

### Sample collection and preparation

The compound microbial strain YSJ was screened and isolated from pig manure–Yanshan ginger organic fertilizer samples. The YSJ compound strains were serially subcultured, and the 10th, 20th, and 30th generations were designated YSJ10, YSJ20, and YSJ30, respectively. Each treatment was replicated three times.

### DNA extraction

Genomic DNA was extracted from the YSJ samples using the E.Z.N.A. Tissue DNA Kit (Omega, USA). The DNA was quantified with a Nanodrop ND-2000 spectrophotometer (Nanodrop, USA). The DNA integrity and size was assessed by 1% (w/v) agarose gel electrophoresis. The DNA samples were stored at −20°C until further use.

### Amplicon library preparation and sequencing

The bacteria were identified by PCR amplification of the V3–V4 region of the *16S* rRNA gene, using the primers 338F (5′-ACTCCTACGGGAGGCAGCA-3′) and 806R (5′-GGACTACHVGGGTWTCTAAT-3′). An 8 bp barcode was added to the 5′ end of the 806R reverse primer for sample differentiation. The reaction volume was 50 μL, which comprised 27 μL ddH_2_O, 2 μL (5 μM) each of the forward and reverse primers, 2.5 μL (10 ng) template DNA, 5 μL (2.5 mM) deoxynucleoside triphosphates, 10 μL of 5× FastPfu buffer, 0.5 μL bovine serum albumin, and 1 μL TransStart FastPfu Polymerase (TransGen, Beijing, China). The amplification protocol comprised predenaturation at 95°C for 5 min, followed by 30 cycles of denaturation at 94°C for 30 s, annealing at 55°C for 35 s, and elongation at 72°C for 30 s, with a final extension step at 72°C for 8 min. The PCR products were purified using a gel recovery kit (Life Technologies, USA), and a Qubit 3.0 fluorometer (Life Technologies) was used for quantitative analysis. The purified amplicons were pooled in equimolar concentrations, and library-specific sequencing adapters were added with the NEBNext Ultra DNA Library Prep Kit (NEB#e7370S/ L) following the manufacturer’s instructions. Dual index sequencing of the paired-end 250 bp reads was performed on an Illumina HiSeq 2500 instrument (Illumina, San Diego, CA, USA).

### Sequence data processing and statistics

The raw reads were filtered using Trimmomatic (v0.36) and Pear (v0.9.6) using the default parameter values to remove low-quality reads. The duplicated reads were merged using Flash (v1.20). The split_libraries_fastq.py script in the QIIME pipeline (v1.8.0) was used to separate the data according to the barcode at the 5′ end of the primer. The sequences were filtered and denoised to remove chimeras, after which they were classified using the Bayesian method against a database derived from the RDP 16S rRNA reference db128. The edited sequences were divided into operational taxonomic units (OTUs) at 97% similarity using Vsearch (v2.7.1). Representative OTU sequences were denoted by the most abundant sequences for each OTU. The OTU table was simplified to the minimum sequence number between samples to directly compare the alpha and beta diversity values of each flora.

### Optimization of culture conditions for YSJ

The degradation rate of the branches and leaves of ginger was determined using the weight loss method [18]. The amount of YSJ used to inoculate the medium was 10%, which was then placed in an incubator at 50°C, and one bottle was removed every day to measure the degradation rate with three replicates. For optimization of the fermentation temperature, the inoculum amount of YSJ was 10%, the temperature was set to 45, 50, 55, and 60°C with three replicates. Based on the optimal culture conditions, the peptone and yeast powder in the peptone cellulose medium were replaced with the corresponding nitrogen sources (peptone, yeast powder, ammonium chloride, and urea), and the remainder of the components were unchanged. The degradation rate was determined after incubation. Peptone + yeast extract powder (ratio of 5:1) was selected as the nitrogen source, and five proportions of 3.0, 6.0, 9.0, 12.0, and 15.0 g/L were then tested with three replicates. The remainder of the components were unchanged and, after inoculation with bacterial liquid, the material was incubated at 55°C and the degradation rate was determined. Based on the optimum culture conditions and nitrogen source, the only carbon source was ginger branches and leaves, for which the tested amounts were 0.5, 1.0, 1.5, 2.0, 2.5, and 3.0 g/L. The remainder of the components were unchanged and, after inoculation with bacterial liquid, the material was incubated at 55°C and the degradation rate was determined with three replicates. Finally, in a 100 mL conical flask, we placed 1 g ginger straw as the carbon source, 5 g peptone and 1 g yeast powder as the nitrogen source, 2 g calcium carbonate, and then added 3.0, 4.0, 5.0, 6.0, or 7.0 g/L sodium chloride. The inoculum amount was 10%, the medium was incubated at 55°C, and the degradation rate was determined.

### Response surface optimization experimental design

Based on the single-factor test results, three factors that had a significant impact on the degradation rate were selected, and the Box–Behnken experimental design was used to optimize the three-factor and three-level response surface [19].

### Statistical analysis

We used a completely randomized block experimental design. Growth values of strains were calculated as the mean of three or four biological replicates per treatment for the in vitro tests. All data were subjected to analysis of variance and a post-hoc least significant difference test for comparison of the means using IBM SPSS Statistics 19.0 software (IBM Corp., Armonk, NY, USA). The significance of differences among the germination data were examined with Student’s *t*-test with a 95% confidence interval and significance level of *p* < 0.05.

## Results

### YSJ microbial community stability and diversity

**Fig 1:**
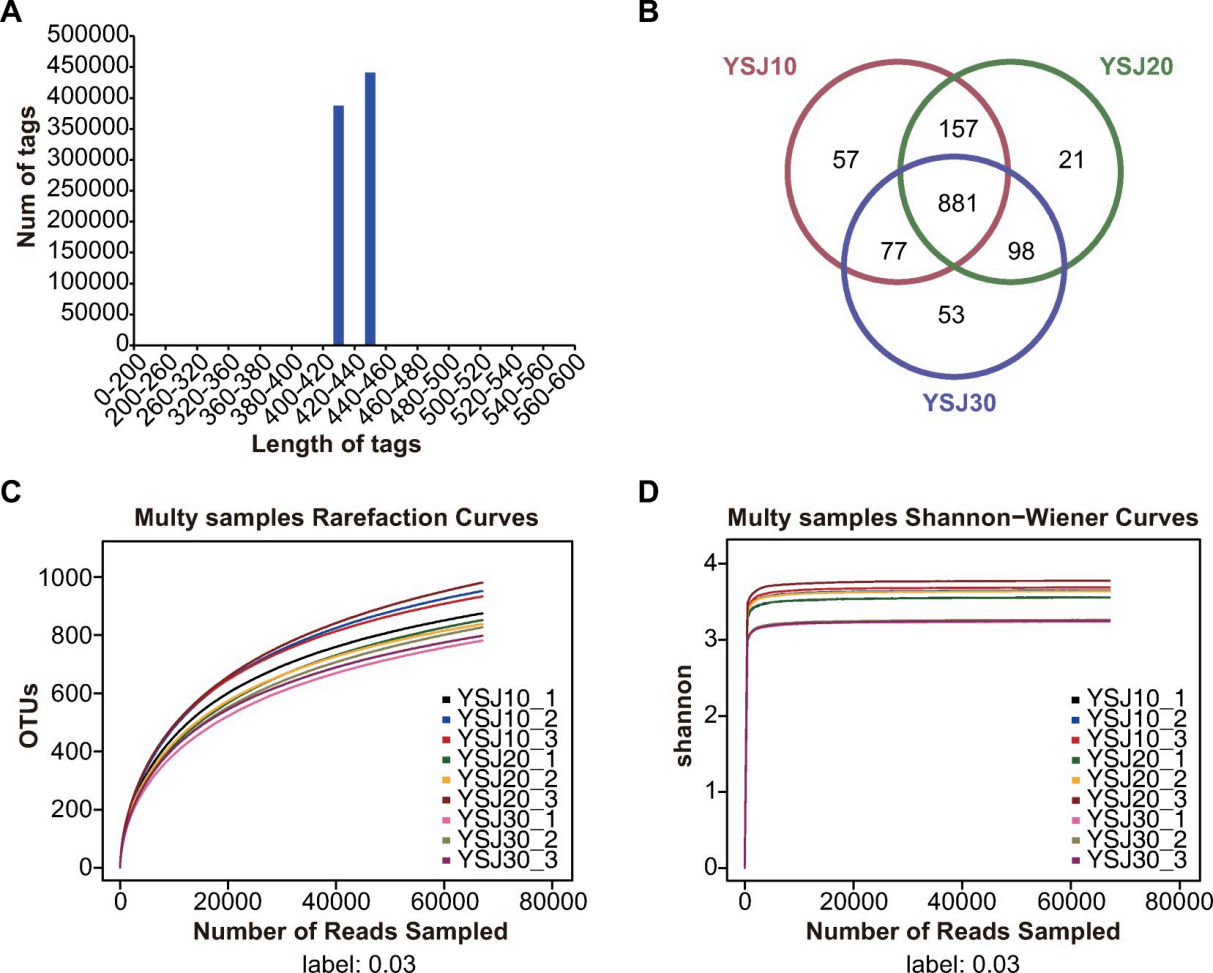
YSJ microbial community stability and diversity. (A) Length distribution of high-quality sequences. (B) Venn diagram of common and unique operational taxonomic units (OTUs) for the YSJ10, YSJ20, and YSJ30 samples. (C) Dilution curves of YSJ detoxification agent samples at the OTU level. (D) Shannon–Wiener index for each sample. The indices were calculated at 97% OTU similarity based on the pyrosequencing data for microbiota in the YSJ samples.

For microbial community composition analysis, 16S rRNA sequencing was conducted. Table 1 shows the number of valid and high-quality sequences obtained for each YSJ sample. The number of valid and high-quality sequences in the YSJ30-2 sample was the smallest among all samples. The proportion of high-quality sequences in each sample was relatively high (>96%). This showed that the valid and high-quality sequences of all samples were of high quality and could be used for subsequent microbial diversity analysis. The lengths of the high-quality sequences obtained by sequencing were mainly concentrated between 420 and 440 bp (Fig 1A).

**Table 1:** 16S rRNA sequencing statistics for the YSJ samples.

Sample number	Valid sequence	High-quality sequence	Proportion
YSJ10-1	76702	74511	97.14%
YSJ10-2	105682	102016	96.53%
YSJ10-3	133938	129467	96.66%
YSJ20-1	114666	110668	96.51%
YSJ20-2	80569	78050	96.87%
YSJ20-3	111215	107600	96.75%
YSJ30-1	85666	82939	96.81%
YSJ30-2	73297	70791	96.58%
YSJ30-3	75164	72422	96.35%

A Venn diagram was generated to visualize the number of shared and unique OTUs for the YSJ10, YSJ20, and YSJ30 samples (Fig 1B). The results showed that YSJ10, YSJ20, and YSJ30 had 1172, 1157, and 1109 OTUs, respectively, with 157 OTUs detected in both YSJ10 and YSJ20, 98 OTUs in both YSJ20 and YSJ30, and 77 OTUs in both YSJ30 and YSJ10. There were 57, 21, and 53 OTUs unique to YSJ10, YSJ20, and YSJ30, respectively. Fig 1C shows the dilution curve for each YSJ detoxification agent sample based on the *S*_obs_ index of OTU richness. The results showed that most of the microorganisms in the YSJ samples were analyzed and the results accurately reflected the composition of the microbial community.

Analysis of the Shannon index curves showed that the samples collected from different subcultures differed in the diversity of their microbial communities. As shown in Fig 1D, the samples of YSJ30-1, YSJ30-2, and YSJ30-3 had low diversity; the samples of YSJ10-1 and YSJ20-1 had moderate diversity; and the samples of YSJ10-2, YSJ10-3, YSJ20-2, and YSJ20-3 had higher diversity. The sample with the highest diversity was YSJ20-3 with a Shannon index of 5.45, whereas the sample with the lowest diversity was YSJ30-1 with a Shannon index of 4.67.

Next, we examined community richness and diversity of microbial ecosystems through alpha diversity analysis (Table 2). The microbial community richness of the nine samples of YSJ was ranked in the following order: YSJ20-3 > YSJ10-2 > YSJ10-3 > YSJ20-1 > YSJ10-1 > YSJ30-2 > YSJ20-2 > YSJ30-3 > YSJ30-1. The Shannon diversity index of the microbial communities in the nine samples was ranked as follows: YSJ20-3 > YSJ10-3 > YSJ10-2 > YSJ20-2 > YSJ10-1, and YSJ20-1 > YSJ30-3 > YSJ30-1 > YSJ30-3. The Simpson diversity index of the microbial communities was ranked as follows: YSJ10-1, YSJ10-2, YSJ10-3, YSJ20-2, YSJ20-3 > YSJ20-1 > YSJ30-1, YSJ30-2, YSJ30-3. By synthesizing the rankings for the Shannon diversity index and Simpson diversity index, the diversity of the microbial communities in the nine samples followed the order YSJ20-3 > YSJ10-3 > YSJ10-2 > YSJ20-2 > YSJ10-1 > YSJ20-1 > YSJ30-3 > YSJ30-1 > YSJ30-3.

**Table 2:** Alpha diversity index statistics for the YSJ samples.

Sample name	Chao1	Goods coverage	OTU	Shannon	Simpson
YSJ10-1	1054.50	1.00	857	5.13	0.93
YSJ10-2	1140.01	1.00	952	5.27	0.93
YSJ10-3	1113.38	1.00	933	5.32	0.93
YSJ20-1	1060.13	1.00	852	5.13	0.91
YSJ20-2	1003.56	1.00	838	5.25	0.93
YSJ20-3	1221.12	1.00	981	5.45	0.93
YSJ30-1	981.04	1.00	781	4.67	0.89
YSJ30-2	1041.11	1.00	827	4.71	0.89
YSJ30-3	986.51	1.00	798	4.69	0.89

Based on the results of the bacterial microbial diversity analysis of the nine YSJ samples, differences in bacterial diversity were observed in samples from different culture generations. For bacterial α-diversity in the YSJ20-3 sample, the microbial chao1 index and Shannon index were the highest among the nine samples, and the number of OTUs was also the largest. The YSJ20-3 sample had the largest number of bacterial species and the highest abundance among all samples. For the YSJ30-1 sample, the chao1 index and Shannon index were the lowest among the nine samples, and the least number of OTUs was detected. Thus, the YSJ30-1 sample had the least number of bacteria and the lowest abundance.

### Taxonomy-based comparison of YSJ samples at the genus level

**Fig 2:**
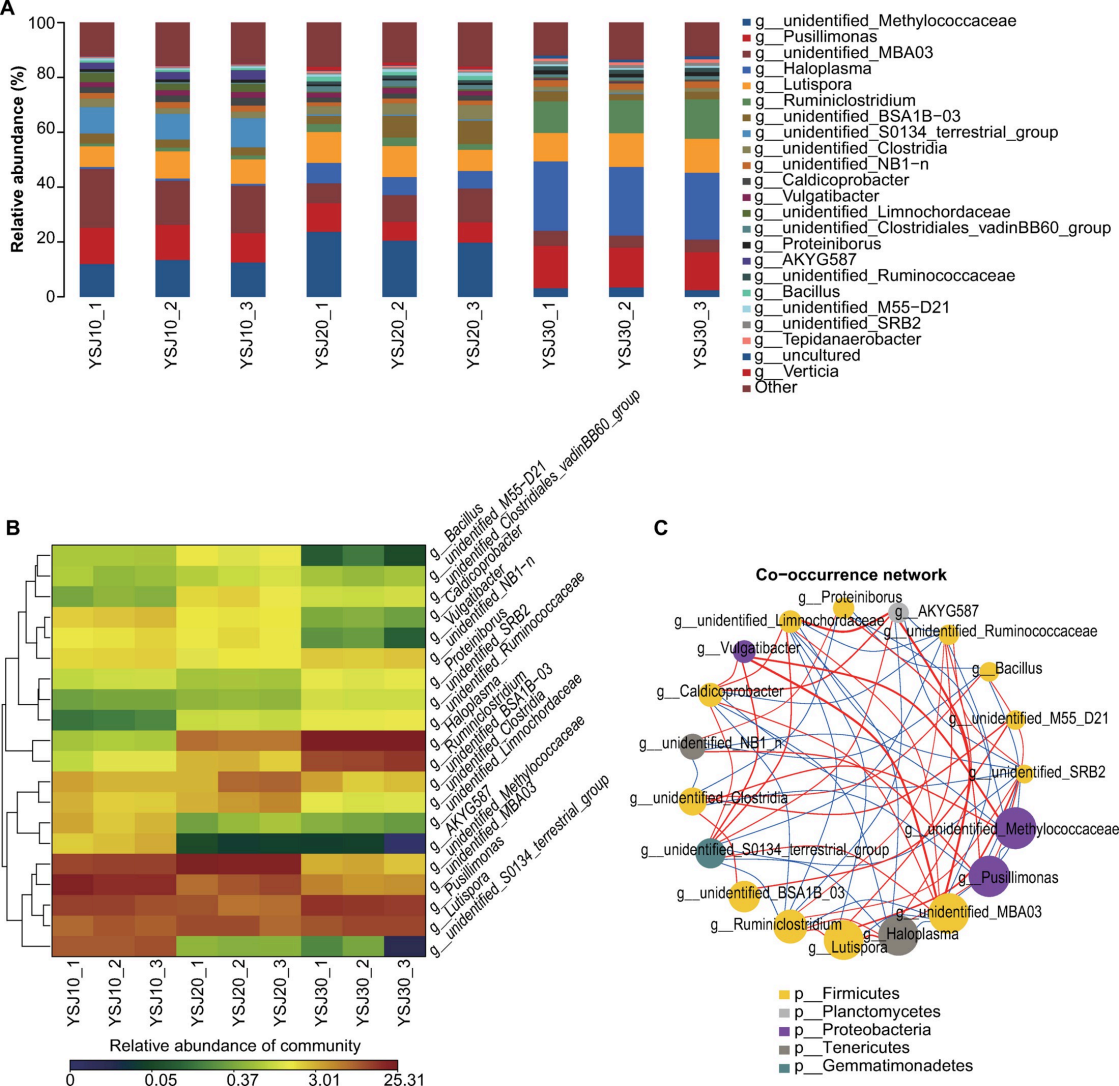
Taxonomic comparison of YSJ samples at the genus level. (A) Relative abundance of the 23 most abundant bacterial genera detected in the YSJ samples. (B) Heatmap of the distribution of the relative abundance of the genera present in the YSJ samples. Columns list genera with relative abundances greater than 1% and are clustered according to phylogenetic relationships. Significance: NS *p* > 0.05, * *p* ≤ 0.05, ** *p* ≤ 0.01. (C) Network of the co-occurring 90% cutoff operational taxonomic units based on correlation analysis. A connection represents a strong and significant (*p*-value < 0.01) correlation. The size of each node is proportional to the number of connections.

Overall microbiota compositions of the 23 most abundant bacterial genera detected in the nine YSJ samples are shown in Fig 2A. The average relative abundance of eight bacterial genera was greater than 1%, namely unidentified Methylococcaceae (2.51%–23.7%), *Pusillimonas* (6.92%–15.4%), unidentified MBA03 (4.36%–21.4%), *Lutispora* (8.5%–12.3%), *Ruminiclostridium* (0.95%–14.4%), unidentified BSA1B-03 (2.31%–3.61%), unidentified Clostridia (1.22%–5.19%), and unidentified NB1-n (1.37%–2.44%). The bacterial genera in the samples collected from different subcultures were notably different.

Among the three samples of YSJ10, the microorganism with the highest relative abundance (21.4%, 16.1%, and 17.2%, respectively) was unidentified MBA03. Among the three samples of YSJ20, the microorganism with the highest relative abundance (23.7%, 20.5%, and 19.7%, respectively) was unidentified Methylococcaceae. Among the three samples of YSJ30, the microorganism with the highest relative abundance (25.3%, 25.1%, and 24.4%, respectively) was *Haloplasma*. Therefore, differences were observed in the dominant microorganisms at the genus level in the YSJ samples collected from different subcultures. *Lutispora* was detected in all nine samples with relative abundance of 8.5%–12.3%, indicating that this genus played a role throughout the process of degradation of Yanshan ginger straw. This finding was consistent with the results of Jiang et al. in a study of cellulose degradation by composite bacteria [20].

The relative abundance of *Ruminiclostridium* increased gradually in the nine samples from YSJ10 to YSJ20 and YSJ30; the abundance in the YSJ30 sample exceeded 10%, which was much higher than that in the YSJ10 and YSJ20 samples. Guo et al. pointed out that *Ruminiclostridium* is a cellulose-degrading bacterium [21], suggesting that YSJ30 has strong cellulose degradation ability. The relative abundance of unidentified S0134 terrestrial group in the three samples of YSJ10 was 9.34%–10.7%, which was much higher than that of the YSJ20 and YSJ30 samples.

To visualize the information for the YSJ samples, we performed a heatmap analysis (Fig 2B). Differences in the composition and relative abundance of the dominant bacterial species in the YSJ samples were apparent.

We further explored the co-occurrence patterns by network inference based on significant and strong correlations using the nonparametric Spearman’s rank correlation analysis [22]. Correlation networks of co-occurring microbes allow visual summarization of large amounts of information [23]. The resulting YSJ microbial network (Fig 2C) consisted of 19 nodes (i.e., OTUs). The co-occurring microorganisms belonged to the Firmicutes, Planctomycetes, Proteobacteria, Tenericates, and Gemmatimonadetes.

There was a positive correlation between the unidentified Methylococcaceae genus and *Vulgatibacter*, indicating that the two genera were highly correlated. The unidentified genus MAB03 was positively correlated with *Vulgatibacter*. The unidentified genera MAB03 and AKYG587, and Limnochordaceae and AKYG587 showed positive correlations.

### Optimization of YSJ culture conditions

**Fig 3:**
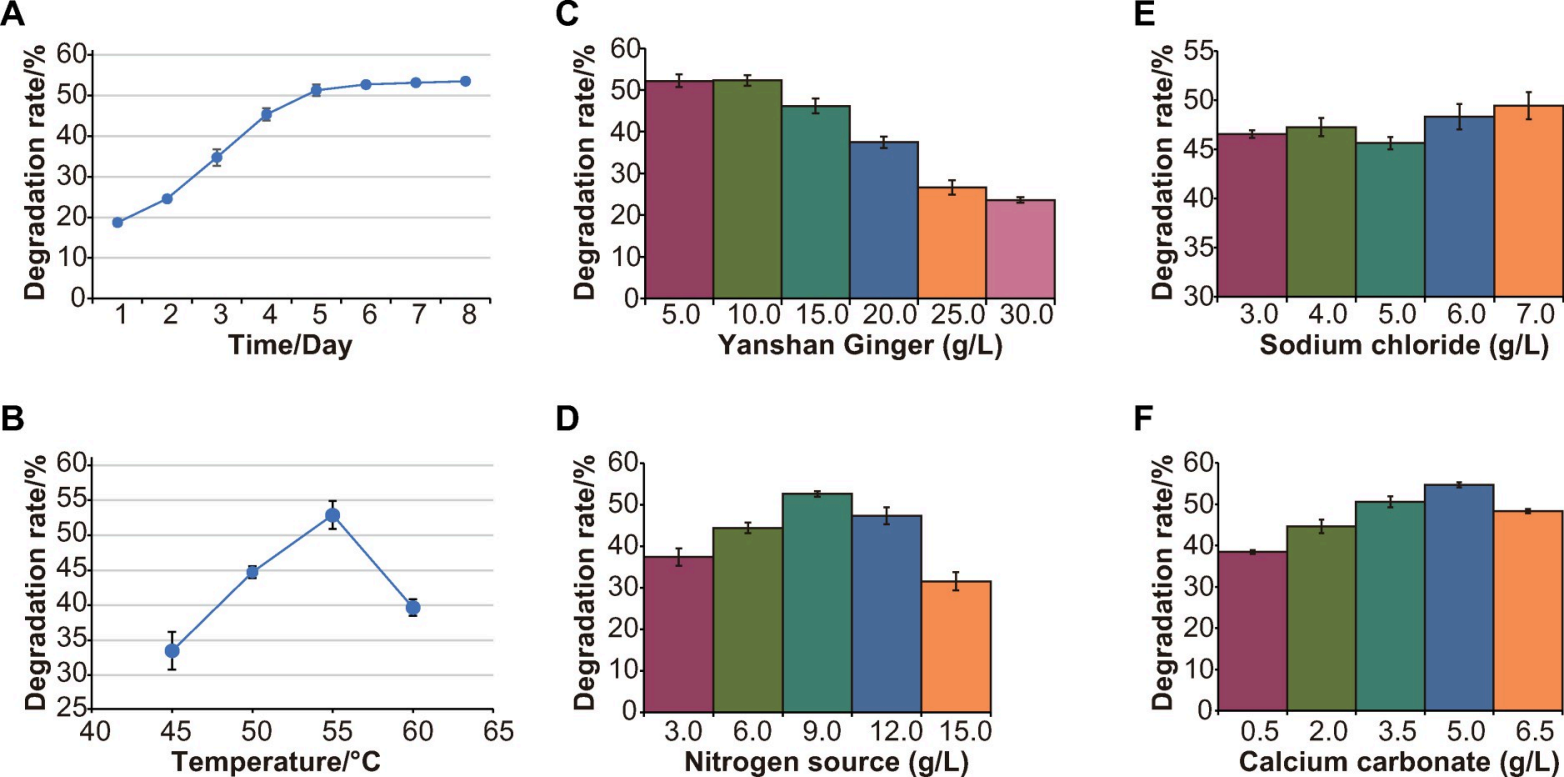
Optimization of YSJ culture conditions. (A) The effect of culture duration on degradation ability of YSJ. (B) The effect of culture temperature on the degradation ability of YSJ (*p* ≤ 0.05). (C) Effect of carbon source addition on YSJ degradation ability (*p* ≤ 0.05). (D) Effect of nitrogen source addition on YSJ degradation ability (*p* ≤ 0.05). (E) Effect of sodium chloride addition on the degradation ability of YSJ. (F) Effect of calcium carbonate addition on YSJ degradation ability (*p* ≤ 0.05).

The degradation rate of Yanshan ginger straw by YSJ showed an increasing trend with extension of the culture duration, peaking on the 6th day and thereafter gradually leveling off (Fig 3A). The final degradation rate was 53.48%. These results indicated that YSJ showed good ability for degradation of branches and leaves of Yanshan ginger.

Different microorganisms have different optimal temperatures for growth. According to the single-factor test results (Fig 3B), the culture temperature had a significant effect on the degradation ability of YSJ in the range of 45–60°C (*p* ≤ 0.05). The degradation rate of Yanshan ginger rose gradually at 45–55°C, peaking at 52.9% at 55°C, and decreased gradually at a temperature higher than 55°C.

Peptone + yeast extract powder, peptone, yeast powder, ammonium chloride, and urea were tested as the only nitrogen sources for screening and optimization of YSJ (Table 3). The effects of the five nitrogen sources on the degradation ability of YSJ differed greatly. Among the nitrogen sources, peptone + yeast extract resulted in the highest degradation rate of 51.69%, whereas the lowest degradation rate was observed with ammonium chloride.

**Table 3:** Effect of nitrogen sources on the degradation ability of YSJ.

Type of nitrogen source	Degradation rate/%
Peptone	45.84±1.00
Yeast extract powder	47.18±2.15
Ammonium chloride	29.41±1.19
Urea	31.73±1.08
Peptone + yeast extract	51.69±0.41

Fig 3C shows that YSJ could degrade different amounts of Yanshan ginger straw. The degradation rate gradually decreased with increased amount of ginger straw, which may be caused by the bioactive substances contained in ginger having an inhibitory effect on YSJ. Addition of ginger material at the rate of 10 g/L resulted in the highest decomposition rate (52.33%).

Based on the test results for nitrogen sources, peptone + yeast extract powder (ratio of 5:1) was selected as the nitrogen source. From Fig 3D, the amount of nitrogen source had a significant effect on the degradation ability of YSJ (*p* ≤ 0.05). When the nitrogen source quantity was 9.0 g/L, the degradation rate was the highest (52.67%).

Inorganic salts are indispensable nutrients for microorganisms and affect their growth and metabolism. Addition of sodium chloride at the rate of 7 g/L resulted in the maximum degradation rate of 49.45% (Fig 3E). The single-factor test (Fig 3F) showed that calcium carbonate had a significant effect on the degradation ability of YSJ (*p* ≤ 0.05). The degradation rate improved with increase in calcium carbonate addition from 0.5 to 5.0 g/L, and the peak value of 54.65% was attained at 5.0 g/L.

### Response surface analysis

**Fig 4:**
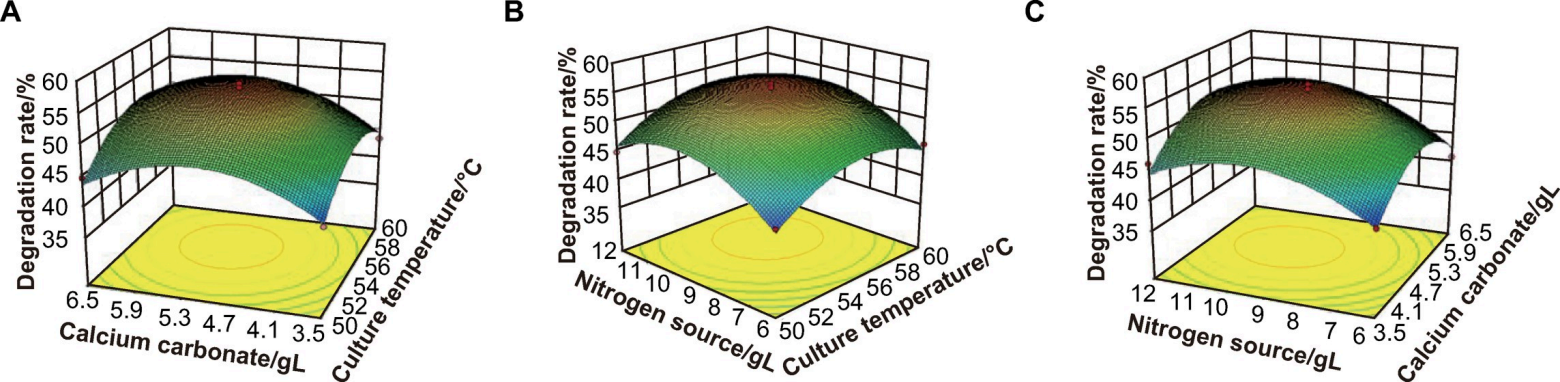
Three-dimensional response surface plots showing the effects of the interaction of culture temperature (A), calcium carbonate addition (B), and nitrogen source addition (C) on degradation rate of Yanshan ginger by YSJ.

Based on the regression equation, the interaction effects of the variables temperature (Fig 4A), calcium carbonate addition (Fig 4B), and nitrogen source addition (Fig 4C) on the degradation rate of Yanshan ginger by YSJ were analyzed by generating a three-dimensional response surface plot. Interactions between variables are significant when the contour plot is elliptical rather than circular. Otherwise, the interaction is trivial. Furthermore, the range of the variables is set reasonably when the shape of the response surface is convex [24].

With the increase in culture temperature and calcium carbonate addition, the degradation of Yanshan ginger rose initially and then decreased when the nitrogen source addition was fixed. The contour line was slightly elliptical and the response surface was convex, revealing that culture temperature and calcium carbonate showed a strong interaction.

With the addition of calcium carbonate at a fixed amount, the degradation rate rose initially and then decreased with the increase in nitrogen source addition and culture temperature. At a temperature of 53–58°C, the degradation rate has an extreme value.

When the culture temperature was fixed, the degradation of Yanshan ginger initially increased and then decreased with increase in calcium carbonate and nitrogen source addition. The response surface was convex and the contour line was slightly elliptical, revealing that the two variables showed a strong interaction and a maximum value.

The derived polynomial equation was analyzed with Design-Expert 10 software, and the optimal culture conditions were determined as follows: culture temperature 56.063°C, calcium carbonate addition 5.241 g/L, and nitrogen source addition 9.746 g/L. Under these conditions, the predicted percentage of Yanshan ginger degradation was 58.768%.

To facilitate the practical operation, the culture conditions for YSJ were revised based on the theoretical values as follows: peptone 8.0 g/L, sodium chloride 9.0 g/L, calcium carbonate 5.2 g/L, yeast powder 1.6 g/L, cellulose (Yanshan ginger branches and leaves) 10.0 g/L, culture temperature 56.1°C, and culture duration 6 d. Under these optimal conditions, the degradation rate for ginger was 58.32%, which was consistent with the value predicted by the model (58.768%) and represented an increase of 14.22% compared with that achieved before optimization.

### Effects of YSJ inoculation on composting of Yanshan ginger

**Fig 5:**
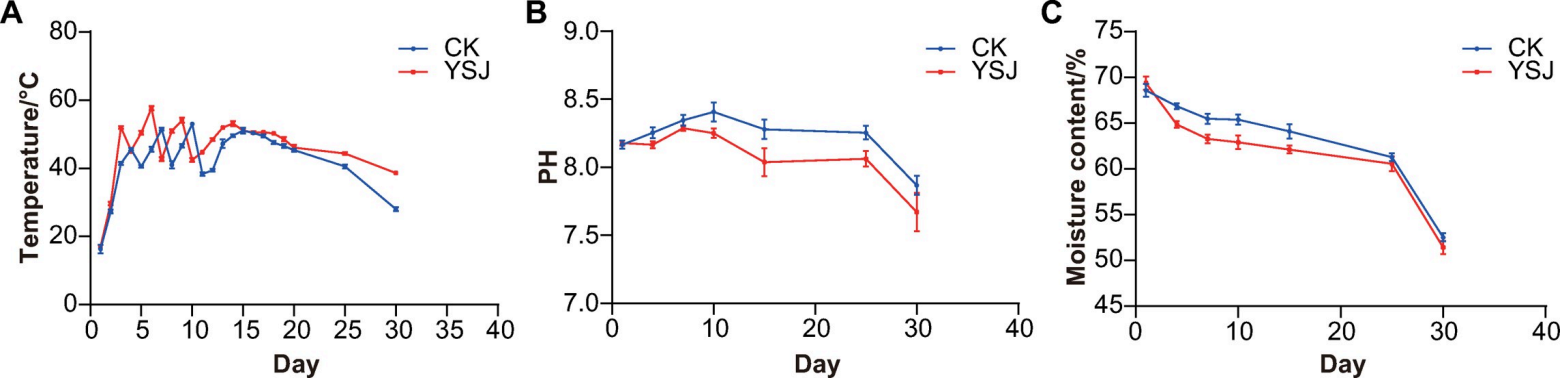
Effects of YSJ inoculation on composting of Yanshan ginger. (A) Changes in temperature during composting. (B) Changes in pH during composting. (C) Changes in moisture content during composting.

Inoculation with YSJ significantly increased the fermentation temperature in the early stage of composting, attaining 52°C on the third day of fermentation, whereas the temperature in the control check (CK) attained 51.5°C on the seventh day (Fig 5A). The high-temperature period under YSJ treatment was significantly longer than that of the CK, possibly caused by the rapid degradation by YSJ of the active substances in ginger that inhibit the growth of microorganisms.

The pH of compost inoculated with YSJ and the CK both peaked (8.26 and 8.40, respectively) on the 10th day of composting (Fig 5B). At the end of composting, the pH was 7.72 and 7.86, respectively. During the entire composting process, the pH of the YSJ treatment was consistently lower than that of the CK.

In the early stage of fermentation, desiccation of the compost inoculated with YSJ was greater than that of the CK (Fig 5C). The moisture content of the compost inoculated with YSJ was lower than that of the CK throughout the process of compost fermentation.

## Discussion

Yanshan ginger is cultivated as a spice and the branches and leaves contain antibacterial substances, such as carvacrol, diethyldimethyl lead, 2,4-di-tert-butylphenol, and borneol [25, 26], which are not conducive to organic fertilizer composting and utilization. The construction of the compound bacterial agent YSJ and optimization of the culture conditions provides a foundation for the production of organic fertilizer derived from Yanshan ginger.

From the single-factor experiment and response surface analysis, we observed that the culture temperature, calcium carbonate addition, and nitrogen source addition have the greatest influence on the ability of YSJ to degrade Yanshan ginger branches and leaves. We determined the optimized culture conditions of YSJ to be as follows: peptone 8.0 g/L, sodium chloride 9.0 g/L, calcium carbonate 5.2 g/L, yeast powder 1.6 g/L, Yanshan ginger branches and leaves 10.0 g/L, incubation temperature 56.1°C, and incubation duration 6 d. Under this optimal condition, the percentage degradation rate of ginger was 58.32%, an increase of 14.22% compared with that of the CK, and consistent with the model-predicted value (58.768%).

Owing to limited time, in the single-factor experiment for selection and optimization of the nitrogen source and inorganic salts in the medium, although a large number of studies were reviewed, easier-to-use and lower-cost raw materials could be selected when combined with the possibility of expanding production in the future [27–29]. In addition, the production of organic fertilizer by composting Yanshan ginger branches and leaves in combination with livestock manure requires further research. The present study of microbial diversity, especially bacteria and fungi in YSJ, has the potential to recognize complex ecosystems of microbe–ginger and microbe–microbe interactions. The major bacterial and fungal genera identified in the YSJ samples have potential for exploration into their use as biological degradation agents.

## Conclusions

The current study confirmed that inoculation of Yanshan ginger branches and leaves with the compound microbial system YSJ increases the fermentation temperature of the compost, prolongs the high-temperature period, and reduces the moisture content and pH of the compost in the initial stage after inoculation. The improvement of these key indices is important to improve the efficiency of composting. In addition, we explored the microbial composition of YSJ using high-throughput sequencing technology, and confirmed that the microbial community was relatively stable and easily subcultured. We optimized the fermentation conditions for YSJ inoculation of Yanshan ginger composting and increased the degradation of Yanshan ginger by 14.22%. These experimental results provide a sound theoretical basis for the practical application of YSJ.
